# A Multicentric Pilot Study of Student Nurse Attitudes and Beliefs toward Sexual Healthcare

**DOI:** 10.3390/healthcare11162238

**Published:** 2023-08-09

**Authors:** Vicki Aaberg, Eva Moncunill-Martínez, Ana Frías, Teresa Carreira, Raquel Fernández Cezar, Alba Martín-Forero Santacruz, Fátima Frade, Daniela Mecugni, Sagrario Gómez-Cantarino

**Affiliations:** 1 School of Health Sciences, Seattle Pacific University, Seattle, WA 98119, USA; aaberv@spu.edu; 2 Paediatric Oncology Unit, Toledo University Hospital, Av. del Río Guadiana, 45007 Toledo, Spain; 3 Faculty of Physiotherapy and Nursing, Toledo Campus, University of Castilla-La Mancha, Avda Carlos III, s/n, 45071 Toledo, Spain; sagrario.gomez@uclm.es; 4 Nursing Department, University of Evora, 7000-811 Evora, Portugal; anafrias@uevora.pt; 5 Superior School of Health, Quinta do Mergulhão Srª da Guia, 2005-075 Santarém, Portugal; teresa.carreira@essaude.ipsantarem.pt; 6 Department of Mathematics, Didactics Area, Faculty of Education of Toledo, University of Castilla-La Mancha, Avda Fábrica de Armas, s/n, 45071 Toledo, Spain; raquel.fcezar@uclm.es; 7 Infanta Cristina University Hospital, Av. 9 de Junio, 2, 28981 Madrid, Spain; albamartinfs@hotmail.com; 8 Departamento de Enfermagem da Criança e do Jovem, Escola Superior de Enfermagem de Lisboa, AvenidabProfessor Egas Moniz, 1600-190 Lisboa, Portugal; fatimafrade4@sapo.pt; 9 Nursing Research, Innovation and Development Centre of Lisbon (CIDNUR), Centre for Public Administration and Public Policies, Institute of Social and Political Sciences, Universidade de Lisboa, 1300-663 Lisboa, Portugal; 10 Campus Universitario San Lazzaro, University of Modena and Reggio Emilia, Vía Amendola, 2, 42122 Reggio Emilia, Italy; 11 Health Sciences Research Unit: Nursing (UICISA: E), Coimbra Nursing School (ESEnfC), 3004-011 Coimbra, Portugal

**Keywords:** sexuality, sexual heath, sexual attitude and belief, nursing, students

## Abstract

Nurse educators are tasked with the education of students to become providers of holistic care, and part of that care includes sexuality. Students carry attitudes and beliefs that influence their behavior; therefore, students who carry negative attitudes about sexual healthcare are less likely to provide that care. This is an international, multicenter study of nursing students’ attitudes and beliefs about the provision of sexual healthcare. The Sexuality Attitudes and Beliefs Survey, which measures attitudes toward the provision of sexual healthcare and has a range of scores from 12 to 72, was given to 129 students across Spain, Portugal, Italy and the United States and revealed negative attitudes about sexuality, with a mean SABS score of 39.95. Higher scores on the SABS reveal more negative attitudes and reduced likelihood of provision of sexual healthcare. Statistically significant differences were found when comparing queer and heterosexual students (41.69 vs. 38.06), and students in their final year of school held more negative attitudes toward the provision of sexual healthcare (41.4 vs. 39.5 and 39.2). This study shows that nurse educators continue to need to focus on the attitudes student nurses carry about sexuality. There is a critical shortage of education strategies to meet the needs of student nurses so that they will be comfortable and confident in providing sexual healthcare.

## 1. Introduction

Sexuality is a complex and personal aspect of health and is an important part of overall health. Sexuality affects every patient, not just some patients within certain sectors of healthcare, and the promotion of sexual health also promotes quality of life. Sexuality is a basic human need, just as food, shelter, clothing, sleep, and rest are necessary for overall health and optimal function. The World Health Organization [1] has defined sexual health as a necessary part of education for healthcare professionals, including for nurses. However, sexual healthcare is often overlooked or omitted from the patient’s care by healthcare professionals [2]. Nurses, who are often the first and most frequent point of contact for patients, must be able to provide care that addresses all aspects of a patient’s health, including their sexual health [2]. 

Nurses are the ideal healthcare professionals to deal with patient sexuality, as they already are knowledgeable about the intimate details of the patient’s functional status, condition, medications and treatment. However, we know that nurses often do not make sexual health a priority in the provision of nursing care and that other nursing responsibilities take higher priority [2,3,4]. While nurses believe that this care is important, the reality is that few provide it [2,3,4,5,6]. While one study revealed that 66% of nurses believed addressing sexuality is part of nursing care, only 13% were familiar with models of how to assess sexuality and almost 80% believed that factors arise that prevent this care [2]. These factors include the belief that sexuality is a taboo subject, that nurses lack both time and knowledge to address sexuality, that they feel shame about sexuality, and that sexuality is not a priority [2]. In order for nurses to address the sexuality concerns of patients, nurses must be educated about sexual health during their nursing education and be willing to devote time to address sexual health during the provision of nursing care. Some nursing schools devote adequate time and importance to teaching about sexual health and others provide little to no education and do not stress the importance of this educational content [7,8,9]. 

It is essential that student nurses have training in holistic care throughout their educational process, and sexuality education is a crucial part of that education [10,11,12,13]. Published literature supports the notion that specific sexuality education in nursing school leads to more positive attitudes about providing sexual healthcare as a nurse [14,15]. Nursing students surveyed about sexuality reported a positive perception of sexual healthcare and were in agreement that the role of the nurse is to provide sexual healthcare [10]. A Turkish study found that students hold conservative attitudes toward sexuality in general and hold more conservative attitudes for themselves than they do for others [11]. Nursing students displayed good knowledge about sexual healthcare and yet were hesitant to provide that care [12]. Students display holding low confidence in their ability to provide sexual healthcare and experience shame in discussions of sexual healthcare [11,13]. 

Nursing student attitudes and beliefs about sexuality have been assessed in studies using the Sexuality Attitudes and Beliefs Survey (SABS). The range of scores possible on the SABS is between 12 and 72, with higher scores indicating stronger negative attitudes and beliefs about sexuality in healthcare and indicates less likelihood that nurses and nursing students will engage in sexual health counseling with patients. Conversely, lower scores indicate more positive attitudes to sexuality and fewer barriers to the provision of sexual healthcare. Previous scores on the SABS have been reported as ranging from a low of 30.62 as part of an experimental study that revealed scores drop to the 22 and 23 range following sexuality education [14] to highs of 44.36 among Jordanian nurses [16] and 47.72 [17] among Chinese nurses and many scores between [15,18,19,20,21,22,23,24,25,26,27].

Several studies across the world have sought to investigate obstacles nursing stu-dents face that inhibit them from providing sexual healthcare to their patients [10,11,12,14,15,18,19,20,22,28,29], but thus far a multicentric collaborative look into the matter has not been conducted. This study reports findings from a collaborative, multicentric project from nursing students in five universities across Spain, Italy, Portugal, and the United States by measuring student nurse attitudes and beliefs about sexuality using the SABS. The multicentric project is part of “EdSex” and is made up of nursing faculty from five universities: The University of Castilla La Mancha, The Polytechnic University of Santarem, The University of Evora, The University of Modena and Reggio-Emilia, and Seattle Pacific University. This EdSex project aims to “train in sexual education to achieve gender equality by empowering women and girls, contribute to reinforcing this in other social spheres, contributing new visions in the field of sexual competence beyond our borders, and to the modernization of sexual education in the socio-health field” [21,30,31,32,33,34,35] and is supported through Erasmus Plus (which is financial support for the education of youth in the European Union). Of note, the researcher from Seattle Pacific University is an invited guest in the project, as the university is outside the European Union.

We hope to improve nursing education curricula in order to help ensure the appropriate provision of sexual healthcare by nurses to all. Through identification of the attitudes and beliefs of nursing students about sexuality, nurse educators will be able to identify curricular changes and pedagogy that will better meet the sexual education needs of students and thus the sexual health needs of patients. The aim of this study is to identify the attitudes and beliefs of nursing students from five universities about sexuality that limit the provision of sexual healthcare.

## 2. Materials and Methods

### 2.1. Design and Sample

This is a descriptive study that represents nursing students from the University of Evora, the Polytechnical Institute of Santarem, the University of Castilla-La Mancha To-ledo, the University of Modena and Reggio Emilia, and Seattle Pacific University. Stu-dents were invited to participate, and data were collected in the fall of 2022 across all lo-cations. Students in Italy study for a total of 3 years to earn a nursing degree and begin the nursing specific courses in their first year. Around 300 students study nursing there at any one time. Students in Spain and Portugal spend 4 years of university study to earn a nursing degree and begin nursing fundamentals along with other perquisite content in their first year. These schools typically enroll between 300 and 350 undergraduate students at a time. In Seattle, there are about 190 undergraduate students who begin nursing courses in their third year of study after completion of prerequisite courses, such as psychology, chemistry, anatomy and physiology, and statistics. These students complete the nursing program in their final 2 years or study out of 4 years of university study. 

Across all five schools, students learn basic anatomy and function of the reproductive system before beginning nursing courses. During the nursing courses they learn about pregnancy, sexually transmitted infections, birth control, sexual dysfunction and the sexual implications of treatments, surgery and medications on sexual function. During clinical experiences they have the opportunity to practice providing patients with sexual healthcare. We invited only students in years 2, 3, and 4 to participate in the study because either students would have had either little or no sexuality content yet or would not have started taking nursing classes; therefore, to survey them without applicable knowledge and experience would be fruitless.

A total of 129 students from the second, third and fourth years of nursing study were recruited through convenience sampling. Data collection included demographic information as well as the SABS. Demographic information collected included age, sex, current year in nursing education, current living situation, sexual orientation, and current sexual partner. Interested students from each university were gathered and informed about the study by faculty members of the EdSex project. Students were informed of the nature of the study, the time commitment to participate and that there were no consequences to not participating. Students interested in participating were provided an online link (written on the board) to the informed consent followed by the study tools, all in a Google document, and classroom space to complete the study.

### 2.2. Sexual Attitude and Belief Survey (SABS)

The SABS was developed as a tool to measure attitudes and beliefs about sexuality held by nurses [22]. The SABS has 12 items and is a self-report scale with Likert-type responses. Participants rate their Likert-type responses from 1= absolutely agree to 6= absolutely disagree. To help avoid acquiescence bias, some of the items are phrased in reverse. The total range of scores possible is between 12 and 72 points, with higher scores indicating stronger negative attitudes and beliefs about sexuality in healthcare and indicates less likelihood that nurses and nursing students will engage in sexual health counseling with patients. Conversely, lower scores indicate fewer barriers to the provision of sexual healthcare. The SABS was reported to have good internal consistency with a Cronbach’s alpha of 0.75 and 0.82 and good test–retest reliability (r = 0.85, *p* < 0.001) [23]. Construct validity is well supported by significant correlation (r = −0.37, *p* < 0.05) with the section of the SKAT (Sexuality Knowledge and Attitudes Test [35]) that measures attitudes toward sexuality.

The SABS was created in English, and this study used the original version as well as versions of the SABS that had been validated for use in Portuguese, Italian, and Spanish. The Portuguese version [25] reported Cronbach’s alphas of 0.72 and 0.80, the Italian version [26] showed a 0.76, and the Spanish version [27] showed a 0.65. These validated versions of the SABS were used in this study based on the dominant language spoken in each country.

### 2.3. Ethical Consideration

This study was approved by the Ethics Committee of Social Research of the Univer-sity of Castilla-La Mancha (CAU-661803-V4Z4). Students were informed of the nature of the research and provided informed consent. Data collection was through an online link. Informed consent was obtained from all participants in the study. No coercion took place; students were willing participants in the research process. 

### 2.4. Statistical Analysis

The data from participants were stored in SPSS program version 28. Data were analyzed using frequencies, percentage distributions, t-tests, and one-way ANOVA, and a *p* < 0.05 was used as signifying a statistically significant result.

## 3. Results

### 3.1. Demographic Information

The complete socio-demographic results are shown in Table 1. The mean age of all participants was 21.8 years. A total of 113 participants (87%) were female. The majority of students (55.8%) were in their third year of education. Approximately half of students (49%) reported they live with their parents currently. A total of 109 (84%) students stated they were heterosexual with 12 (9%) students reporting they were bisexual and 5 (3.9%) reporting they were homosexual. Of note, the bisexual, homosexual, and not defined orientation participants will be grouped together as ‘queer’ since the numbers are small. A majority (61%) of students reported they currently have one unique sexual partner, and 6% reported they have never had a sexual partner.

### 3.2. SABS Scores by School

The mean overall SABS score as well as mean SABS scores by university are reported in full in Table 2. The mean SABS for all participants (*n* = 129) was 39.95. The range of scores by university was a low mean of 36.27 from Modena and a high mean of 44.15 from Santarem. Broad consistency was noted across all schools. The questions that showed the lowest barriers to students was ‘Sexuality is too private an issue to discuss with patients’ with a mean of 1.83 and ‘Whenever patients ask me a sexually related question, I advise them to discuss the matter with their physician’ with a mean of 2.37. The questions with the greatest barriers identified in this study are ‘I understand how my patient’s diseases and treatments might affect their sexuality’ with a mean of 5.05 and ‘Discussing sexuality is essential to patients’ health outcomes’ with a mean of 4.78. 

### 3.3. SABS Scores by Demographic Characteristics

The SABS scores by demographic characteristics are displayed in Table 3. Female students demonstrate a lower SABS score (38.12) than males (41.25), with a non-significant result (t = 1.91, *p* = 0.06). In addition, heterosexual students revealed a lower mean SABS (38.06) than queer students (41.69) with statistical significance (t = 2.30, *p* = 0.02). Other characteristics (current sexual partner status, year in school, and current residence) did not generate statistically significant results.

## 4. Discussion

Attention to patients’ sexual healthcare is critical for nurses, and sexuality topics are regularly neglected by nurses [2,3,4]. Nurses have low confidence and comfort in dealing with sexual healthcare and require education to support their ability to provide this care [3,4,5,6,13,16,17,23,24]. Nurses who focus their continuing education on sexuality may improve their ability to provide sexual healthcare. However, since all practicing nurses go through the educational system, the focus for this study was to assess the attitudes and beliefs of nursing students in the provision of sexual healthcare in an attempt to identify barriers to care. Once these barriers are identified, nursing faculty can work to update curricular requirements to better meet the needs of student nurses and thus contribute to better meeting patient needs for sexual healthcare.

The mean SABS score for this study of 39.95 is comparable to other published reports of nursing student SABS results and indicates more negative attitudes toward sexuality. The lowest mean previously reported in the literature was 30.62 [14]. Other SABS means with nursing students reported in the mid-range have been 32.54, 32.38, 36.69, and 38.08 [15,18,19,26]. The higher end of means with nursing students have been reported as 41.55 and 42.3 [15,18]. The results of SABS have been reported with practicing nurses with a broader range of results. US nurses have demonstrated SABS means of 32.24 and 33.7 [24,29]. Jordanian nurses have reported a mean of 44.36, and Chinese nurses have reported a mean of 47.72 [16,17]. Differing attitudes and beliefs about sexuality may be traced to varying social and cultural norms, yet providing holistic care is a goal of nurses worldwide. The SABS scores revealed in this study point to a higher risk that sexual healthcare, and so the quality of life of patients, will be compromised if these soon-to-be-practicing students do not address sexual healthcare. 

The United Nations has established a list of Sustainable Development Goals. Goal 3.7 specifically focuses on sexual health and states: “By 2030, ensure universal access to sexual and reproductive health-care services, including for family planning, information and education, and the integration of reproductive health into national strategies and programmes” [36,37]. It is clear that there is a global need for sexuality education, especially for future healthcare providers. Nursing education and the social and cultural norms around sexuality vary between Portugal, Spain, Italy, and the United States. In Portugal in 2009, a law mandating sexual education was established. This law requires sexual and effective education with a focus on reproduction, orientation and sexual violence to reduce risk among youth [38]. In Italy, each school for children 6–18 years of age decides whether to teach sexuality content and if so, what to teach. Previous research documents that 37% of Italian youth reported they learned sexuality from their parents or teachers, 25% from friends, 16% from books, 15% from the internet, and the others from unspecified sources [39]. Clearly, many Italian youth may not receive adequate sexual education. In Spain, federal law states that sexual education must be part of education of youth and specifically states that the educational formation of healthcare professionals must include curricular content about sexual and reproductive health [40]. In the United States, each of the 50 states decides whether to require sex education for 5–18-year-olds, and many conservative states provide simply abstinence-only education. Washington state (the location of Seattle Pacific University) began to require comprehensive sexuality education in public schools in the 2022–2023 school year [41], yet these participants were already at the university. No specific regulation exists in the US that mandates sexuality education in nursing curricula.

The results from this study show that the highest barriers to the provision of sexual healthcare were from students in Santarem, and the lowest barriers were from students in Italy. The one-way ANOVA revealed (F = 3.16, *p* = 0.016) significant statistical difference between at least two groups. Tukey HSD for multiple comparison showed that the mean score was significantly different between Modena and Santarem (*p* = 0.021) as well as between Modena and Seattle (*p* = 0.033). Other significant comparisons between schools were not found. These differences may be explained by cultural norms around sexuality. Italian students showed the highest likelihood to provide sexual healthcare despite inconsistent early childhood sexuality education. Students in Santarem had a much higher SABS mean than other Portuguese students from Evora. This difference may be explained by the regular focus on sexuality in the nursing program in Evora. Historically, there have been sexuality-based programs and workshops that are not a required part of the curriculum but students are invited to attend to enhance their education [42]. The students from Seattle Pacific University were the only participants to attend a faith-based private university. The university may attract students with more conservative beliefs, which may lead to stronger barriers against open discussions about sexuality and acceptance of diverse sexual orientation and gender identity, which may result in less likelihood of sexuality being discussed in the provision of healthcare. Overall, nursing education in these countries emphasizes the importance of sexual and reproductive health and the prevention of STIs [7,8,9]. However, the depth and breadth of sexuality education in nursing programs can vary among specific programs and countries. Some nursing programs may have a more conservative approach to sexuality education, while others may include more comprehensive education about sexual and reproductive health and issues related to sexual diversity [7,8,9]. Overall, our students continue to hold negative attitudes and are uncomfortable discussing sexuality. 

The lowest barrier to providing sexual healthcare identified in this study was revealed in the students’ responses to ‘sexuality is too private to discuss’ with a mean score of 1.83. This result is in contrast with other reports of the SABS with nursing students. Other studies reported that student responses to this question showed a mean of 3.10 [20], 3.85 [18], 3.66 [28] and 4.04 [15]. The question with the second lowest barrier reported in this study was ‘Whenever patients ask me a sexually related question, I advise them to discuss the matter with their physician’ with a mean of 2.37. This result has previously been reported with means of 3.30 [18], a 2.18 [20], a 2.77 [28], and a 2.66 [15]. While the differences in mean scores between this study and previously reported studies are significant, they can likely be explained by differences in social structure and expectations around sexuality in various countries.

The greatest barrier to the provision of sexual healthcare in this study lies in students’ estimation of their understanding of how the patient’s disease and treatments might affect their sexuality, with a mean of 5.05. Other studies reported their students to have a mean of 2.51 [20], 3.10 [18], 3.00 [28], and 4.27 [15]. The next greatest barrier was the low belief that discussing sexuality is important to patient’s health outcomes, with a mean of 4.78. In contrast, other reported means have been 1.79 [20], 2.98 [18], 2.66 [28], and 1.68 [15]. Significant differences exist between the results of this study and other published reports. Again, these differences may reflect cultural and social norms around discussions of sexuality [38,39,40]. Another explanation for the high barrier here is that 68% of students were in their second or third year of nursing school and possibly there had been little sexuality content at this point in their education [7,8,9]. The study that has reported the lowest mean on this question of a 1.79 [20] focused exclusively on students in their final year of nursing school. Regardless of variations between current and previously reported SABS scores, it is troubling that nursing students do not have a strong understanding of how a patient’s disease and treatment may affect their sexuality and that students in this study do not believe that discussions of sexuality are important to patient health outcomes. Curricular emphasis on the impact of diseases and treatment on sexuality may be an effective means of breaking down this highest barrier [14,15].

The question resulting in the third highest score from this study is student responses to ‘giving a patient permission to talk about sexual concerns is a nursing responsibility’, with a mean score of 4.68. This result indicates that nursing students prefer to avoid discussion of sexuality altogether. Nurses spend more time with patients than other healthcare providers and are the obvious professionals to have these discussions. Physicians spend limited time with patients, and patients may feel intimidated to ask them about sexuality. However, nurses may not have the information needed to deal with sexual health issues [2,3,4,5,6]. Nurses have been discussing these issues for decades. In nursing education in the 1970s and 1980s, there was a focus on the critical nature of the provision of sexual healthcare by nurses [31,32,33,34], and in the 1990s discussions were around the practice of healthcare providers waiting for the patients to ask about sexual functioning and then provide information [32,33]. We continue to discuss these same issues 4 and 5 decades later and have seemingly made very little progress. If nursing students do not feel it is their role to give permission to patients to talk about sexuality, then who will provide this vital care?

Comparison of the results between male and female participants in this study showed no statistically significant difference, with male students (*n* = 16) holding stronger barriers to the provision of sexual healthcare than females (41.25 vs. 38.12, *p* = 0.06). Other results were reported previously, with males revealing a mean of 34.54 and females 32.22 [20] and with males showing 40.21 and females 37.28 [28]. While these results are not statistically significant, male nursing students may face more barriers to addressing sexuality with patients than their female counterparts due to societal norms and gender roles. In many cultures, men are socialized to be less expressive emotionally around intimate issues, which can make it challenging for male nursing students to initiate conversations about sensitive topics, such as sexual health [19,28]. Additionally, patients may feel more comfortable discussing intimate topics with nurses who share the same gender, and male nursing students may face resistance or discomfort from patients who prefer to work with female nurses [19]. This lived experience may make it less likely that male students will provide comprehensive care to patients to address their sexual health needs. It is possible also that males working in nursing may be very aware of the intimate nature of some parts of nursing care and thus are hesitant to be perceived as sexually aggressive or giving any impression of themselves as sexual beings to spare discomfort in their patients [19,28]. The difference between male and female nurses and nursing students is a clear direction for further study with larger samples that has been revealed here. 

Another statistically significant difference in mean SABS scores came from student sexual orientation. While the number of students who reported they were queer (homosexual, bisexual, or undefined) was not large (*n* = 17), they had a higher SABS mean (41.69) than students who reported they were heterosexual (38.06, t = 2.30, *p* = 0.02). No other published studies have reported this phenomenon. Queer nursing students may face unique barriers in providing sexual healthcare to patients due to systemic discrimination and stigma towards LGBTQ+ individuals in healthcare [43]. They may also experience discrimination and bias from colleagues or patients, which can impact their confidence in providing care and addressing sexuality-related issues with patients [43]. Additionally, healthcare systems may not provide adequate training on LGBTQ+ healthcare [7,8,9] or may lack resources to support queer patients, which can make it difficult for queer nursing students to provide comprehensive and affirming care. Since the average age of participants in this study was 21.8, possibly the queer students are mostly in the developmental stage of becoming confident about their sexual orientation or identity and therefore have more hesitation about addressing patient sexuality concerns. The differences in attitudes about sexual healthcare between heterosexual and queer individual calls for further study with larger samples.

Sexuality is a topic of significant stigma and involves values and morality. Young people’s lack of exposure to open conversations about sexuality in their families may be tied to family values and moral judgement about behaviors, specifically about sexuality. These values and judgments may cause some student nurses to be uncomfortable speaking openly about sexuality. Other student nurses may have been raised in families in which sexuality was a taboo topic. It follows, then, that some student nurses may be uncomfortable discussing sexuality with anyone and do not feel confident in their ability to do so, and this results in some patients not receiving the information needed to attend to their sexual health [7,8,9,15,19]. This is striking given that student nurses ask patients about other intimate aspects of their lives, such as bowel habits. While nursing faculty cannot prevent negative attitudes about providing sexual healthcare, they can address this in the classroom and clinical settings by requiring students to practice providing sexual healthcare and completing written reflections about their experiences.

Despite an unbalanced sample (more students from Spain and comparatively few from Italy, where the lower number reflects students in one elective sexuality course with few enrollees), there is remarkable consistency from school to school around not feeling comfortable discussing sexuality, believing these discussions are not the role of the RN, deferring sexuality-based questions to the provider, and assuming patients are too sick to be interested in sexuality. These results all point to the lack of focus and attention given to sexuality education in nursing education. Nurse educators, however, are perfectly positioned to make some serious changes in nursing education to benefit patients.

Nursing faculty attitudes and beliefs about sexuality, their level of comfort with discussing sexuality, and their likelihood to discuss sexuality related to health concerns were not measured in this study, yet these factors likely impact the quality of student preparation to provide sexual healthcare. The literature has documented some of the limits of sexuality education in nursing schools [7,8,9]. One study from the UK documented sexuality content in nursing programs there and summarized that nurses are “poor at sexuality”, that sexuality is considered a taboo subject still or is regularly ignored in favor of other nursing topics and that some faculty are not comfortable discussing sexuality [8]. A separate study from the US revealed that 27% of the programs reported no sexuality content in the curriculum, some reported having no LGBTQ content or had students practice taking sexual history and found that faculty are not comfortable with discussing sexuality [7]. Another study correlated nursing faculty characteristics and reported that faculty who are older, have expertise in women’s health content, are PhD prepared, and have extensive nursing practice and teaching experience have lower SABS scores [36]. 

Nursing faculty have the central role in the education of nursing students about sexuality and have control over the curriculum and teaching strategies. It is incumbent on faculty who lack knowledge in sexuality to become educated to teach this content. Teaching strategies to enhance sexuality education include the following: (1) Create an open dialogue. It is important for nursing students to feel comfortable discussing sexual health topics in class. Creating an open dialogue by requiring active participation in the classroom and providing a safe space for students to ask questions can help them gain a better understanding of the material. (2) Utilize interactive teaching methods. Interactive teaching methods, such as problem-based learning, case studies, and simulations can be used to help nursing students understand and apply the knowledge they gain in the classroom to real-world scenarios. These interactive methods practiced in the classroom should also help increase comfort and confidence in discussing sexuality and lead to improved provision of sexual healthcare. (3) Incorporate hands-on clinical practice. Clinical practice is an important component of any nursing program, and it is particularly important when it comes to providing sexual healthcare. Providing nursing students with supervised clinical experiences and feedback that involve sexual healthcare can help them develop the necessary skills to become competent nurses in this area. Future research should include the efficacy of teaching strategies such as these to improve student nurse attitudes toward the provision of sexual healthcare.

Failure to provide sexual healthcare can have serious public health implications. Limited access to sexual healthcare may result in limited access to screenings for sexually transmitted infections (STIs) and to preventative care such as contraception. Patients may leave the hospital unaware of the sexual side effects of new medications that have been prescribed or limitations following treatment or surgery that impact their sexual function or sexual health. This can lead to an increase in STI rates, unintended pregnancies, and other health problems. Furthermore, people who do not have access to sexual healthcare may be at a greater risk of engaging in risky behaviors. Finally, without access to sexual healthcare, individuals may be unable to receive support and advice to help them make informed decisions about their sexual health.

A limitation of this study is the sample size of 129 participants. Additionally, the small number of students from each school could represent selection bias, and the disproportionate number of students (one school with 12 and another with 47) leads to unbalanced participant information. More similar numbers of participants would provide better representation of students across the five schools. We did not ask participants to identify their gender identity and therefore missed the opportunity to collect data about trans or non-binary individuals. While the aim of this study did not include measuring faculty attitudes and beliefs about sexuality, this information would lead to a more comprehensive and robust discussion about the issues surrounding the education of nursing students about sexuality. Future studies with a larger number of participants, more male nursing students, including gender identity in demographic information, and more balanced numbers between universities would lead to stronger findings.

## 5. Conclusions

This study clearly shows that these nursing students hold attitudes and beliefs that may interfere with the provision of sexual healthcare. Sexual health is an integral part of overall health, and nurses need to be knowledgeable about common sexual health concerns such as sexually transmitted infections (STIs), contraception, and sexual dysfunction in order to assess, diagnose, and treat these conditions. The attitudes held by students, who are the next generation of nurses, may severely limit their behaviors to provide this care. Nurse educators have the responsibility to take steps in the direction of making sure students understand disease processes and treatments that may affect sexuality, and we can also increase focus on sexual health needs to improve holistic health outcomes. Nurse educators can engage these barriers and promote comfort and confidence by requiring students to practice giving patients permission to discuss sexual health concerns, either in the clinical setting or through simulation or role play in the classroom. These measures may result in more positive attitudes about sexuality in nursing students that may lead to better provision of sexual healthcare. 

## Figures and Tables

**Table 1:** Socio-demographic characteristics of students.

Characteristics	*n*	%	Mean (SD)
Total *n* = 129			
**Gender**			
Female	113	87.6	
Male	16	12.4	
**Age** (mean)			21.8 (1.6)
Range 18–37 years			
**Year of nursing study**			
Second	28	21.7	
Third	72	55.8	
Fourth	29	22.4	
**Current Residence**			
With parents	63	49	
Rented room	12	9.2	
University residence	13	10	
Rental with colleagues	36	30	
Other	5	3.8	
**Sexual Orientation**			
Heterosexual	109	84.5	
Bisexual	12	9.3	
Homosexual	5	3.9	
Undetermined	3	2.3	
**Sexual partner status**			
One unique partner	79	61.2	
No partner now	40	31.2	
Never had sexual partner	8	6	
More than 1 sexual partner	2	1.5	

**Table 2:** SABS scores by survey item and school, ANOVA of school means.

Survey Items	Evora	Santarem	Castilla	Modena	Seattle	Mean
	Portugal	Portugal	Spain	Italy	USA	
	N = 29	N = 21	N = 47	N = 12	N = 20	
Discussing sexuality is essential to patients’ health outcomes						
	5.00	5.45	4.72	4.09	5.10	4.78
2. I understand how my patient’s diseases and treatments might affect their sexuality						
	5.40	5.35	4.87	4.91	4.70	5.05
3. I am uncomfortable talking about sexual issues						
	2.35	2.95	2.82	2.91	3.55	2.92
4. I am more comfortable talking about sexuality issues than most of the nurses I work with						
	2.85	3.55	3.10	3.18	2.70	3.08
5. Most hospitalized patients are too sick to be interested in sexuality						
	2.42	3.15	2.46	2.45	2.35	2.57
6. I make time to discuss sexual concerns with my patients						
	3.35	3.05	2.41	2.55	2.80	2.83
7. Whenever patients ask me a sexually related question, I advise them to discuss the matter with their physician						
	1.89	2.85	2.54	2.00	2.55	2.37
8. I feel confident in my ability to address patients’ sexual concerns						
	3.89	3.85	3.48	4.55	3.80	3.91
9. Sexuality is too private an issue to discuss with patients						
	1.64	2.10	1.91	1.54	1.95	1.83
10. Giving a patient permission to talk about sexual concerns is a nursing responsibility						
	5.10	5.55	4.74	3.09	4.90	4.68
11. Sexuality should be discussed only if initiated by the patient						
	2.53	2.75	2.22	2.27	3.55	2.66
12. Patients expect nurses to ask about their sexual concerns						
	3.36	3.55	3.15	2.73	3.60	3.28
13. Mean SABS Score by school						
	39.82	44.15	38.43	36.27	41.42	
Mean SABS Score Total Sample						
	N = 129	Mean SABS = 39.95				
	ANOVA of School means F(4, 124) = 3.15, *p* = 0.016					

All Likert-type responses for SABS 1–6, range of SABS total 12–72. Lower score indicates more positive attitude and fewer barriers to the provision of care.

**Table 3:** SABS scores by demographic characteristics.

Characteristics	*n*	Mean (SD)	Range	Statistics	Effect Size
Males	16	41.25 (6.81)	32–56	t = 1.91	
Females	113	38.12 (6.03)	19–66	*p* = 0.06	d = 0.45
Queer	17	41.69 (6.97)	19–66	t = 2.30	
Heterosexual	109	38.06 (5.92)	24–55	*p* = 0.02	d = 0.52
Never had sexual partner	8	39.13 (6.45)	19–42	t = 0.28	
Had sexual partner	121	38.47 (6.51)	24–66	*p* = 0.23	d = 0.10
Year in school					
2	33	39.2 (4.7)			
3	57	39.5 (8.6)		f = 0.98	
4	39	41.4 (7.2)		*p* = 0.37	n^2^ = 0.022
Current residence					
With parents	58	38.9 (6.1)			
Rental with colleagues	37	39.1 (7.8)			
University housing	15	43.2 (10.4)			
Rented room	5	46.2 (12.4)		f = 2.05	
Other	14	41.1 (3.3)		*p* = 0.09	n^2^ = 0.041

## Data Availability

Not applicable.
